# RETRACTION: Hypoglycemic, Anti‐Inflammatory, and Neuroprotective Potentials of Crude Methanolic Extract From *Acacia Nilotica* L. – Results of an In Vitro Study

**DOI:** 10.1002/fsn3.72358

**Published:** 2026-09-22

## Abstract

**RETRACTION**: A. Rauf, M. Ibrahim, T. S. Alomar, N. AlMasoud, A. A. Khalil, M. Khan, A. Khalid, M. S. Jan, D. Formanowicz and M. M. Quradha, “Hypoglycemic, Anti‐Inflammatory, and Neuroprotective Potentials of Crude Methanolic Extract From *Acacia Nilotica* L. – Results of an In Vitro Study,” *Food Science & Nutrition* 12, no. 5 (2024): 3483‐3491, https://doi.org/10.1002/fsn3.4017.

The above article, published online on 13 February 2024 in Wiley Online Library (wileyonlinelibrary.com), has been retracted by agreement between the journal Editor‐in‐Chief, Y. Martin Lo; and Wiley Periodicals LLC. The retraction has been agreed upon following concerns raised by a third party. An investigation identified inconsistencies in Table 3 where the authors claimed that inhibition increased in a concentration‐dependent manner. However, the values shown in the table are not consistent with the reported trend. Additionally, concerns were raised regarding the reported ± SEM values in Tables 1–3, including one value reported with no apparent variation. The article reports results as mean ± SEM but does not specify the number of replicates or independent experiments used to calculate these values.

The authors cooperated with the investigation and explained that two values in Table 3 had been inadvertently transposed. However, they were unable to provide the underlying raw data to support this explanation or to verify that the reported values were otherwise correct. As the underlying replicate data was not available, neither the reported trend nor the ± SEM values could be independently verified. As a result, the editor considers the results and conclusions of this article to be unreliable. The authors disagree with the retraction.

**RETRACTION**: A. Rauf, M. Ibrahim, T. S. Alomar, N. AlMasoud, A. A. Khalil, M. Khan, A. Khalid, M. S. Jan, D. Formanowicz and M. M. Quradha, “Hypoglycemic, Anti‐Inflammatory, and Neuroprotective Potentials of Crude Methanolic Extract From *Acacia Nilotica* L. – Results of an In Vitro Study,” *Food Science & Nutrition* 12, no. 5 (2024): 3483‐3491, https://doi.org/10.1002/fsn3.4017.

The above article, published online on 13 February 2024 in Wiley Online Library (wileyonlinelibrary.com), has been retracted by agreement between the journal Editor‐in‐Chief, Y. Martin Lo; and Wiley Periodicals LLC. The retraction has been agreed upon following concerns raised by a third party. An investigation identified inconsistencies in Table 3 where the authors claimed that inhibition increased in a concentration‐dependent manner. However, the values shown in the table are not consistent with the reported trend. Additionally, concerns were raised regarding the reported ± SEM values in Tables 1–3, including one value reported with no apparent variation. The article reports results as mean ± SEM but does not specify the number of replicates or independent experiments used to calculate these values.

The authors cooperated with the investigation and explained that two values in Table 3 had been inadvertently transposed. However, they were unable to provide the underlying raw data to support this explanation or to verify that the reported values were otherwise correct. As the underlying replicate data was not available, neither the reported trend nor the ± SEM values could be independently verified. As a result, the editor considers the results and conclusions of this article to be unreliable. The authors disagree with the retraction.

